# Multiple endocrine glands insufficiency due to langerhans cell histiocytosis (LCH): a case report

**DOI:** 10.1186/1687-9856-2015-S1-P119

**Published:** 2015-04-28

**Authors:** DoThi Thanh Mai, Vu Chi Dung, Bui Phuong Thao, Nguyen Ngoc Khanh, Can Thi Bich Ngoc, Nguyen Phu Dat

**Affiliations:** 1 National Hospital of Pediatrics, Hanoi, Vietnam

## 

LCH is the rare disease involving clonal proliferation of Langerhans cells, abnormal cells deriving from bone marrow and capable of migrating from skin to lymph nodes. The clinical presentation of LCH may occur in multiple organs: bone, skin, lymph nodes or pituitary gland, but clinical presentation of LCH rarely occurs in multiple endocrine systems.

We presented a special case who was diagnosed with LCH and presentation of LCH occurred in multiple systems: pituitary gland, thyroid, adrenal gland.

A 11 years old girl was hospitalized for lump on the neck. Past history: one year ago, she was diagnosed with autoimmune polyendocrine syndromes and treated with hormone replacement (levothyroxine 3mcg/kg/day, desmopressin 4 mcg/kg/day, hydrocortisone 10 mcg/kg/day). Physical examination showed: she had a swollen lump on her neck, she had a temperature of 38° C. She had no polyuria, no polydipsia. Her height was 141cm (-0.29 SD); her weight was 37 kg; her BMI was 18.5 (50th – 70th) .She had normal growth velocity and normal pubertal development. Laboratory evaluation revealed : WBC : 10.3 × 109 /l ( normal range : 4×10 9 /l – 10 ×109 / l) ; CRP : 105.78 mg/l ( normal range : < 10 mg/l ); serum cortisol at 8 a.m : 16.3 nmol/l ( normal range : 200 – 600 nmol/l ); T3 : 1.8 nmol/l ( normal range : 1 – 3 nmol/l ), T4 : 135.9 nmol/l ( normal range : 50 – 150 nmol/l ), TSH : 0.002 mUI/ml ( normal range : 1 – 5 mUI/ml ); blood osmotic pressure : 279 moms/kg, urinary osmotic pressure : 127 mosm/kg; plasma glucose level and electrolyte were normal. An MRI of brain showed: thickened pituitary stalk. A biopsy of the lump on her neck showed: features of Langerhans Cell; skeletal and long bone radiograph showed no osteolytic lesion.

She was treated with hormone replacement and chemotherapy.

Written informed consent was obtained from the patient's parent or guardian for publication of this Case report (and any accompanying images). A copy of the written consent is available for review by the Editor-in-Chief of this journal.

